# Breast Cancer Treatment: To tARget or Not? That Is the Question

**DOI:** 10.3390/cancers15235664

**Published:** 2023-11-30

**Authors:** Alexandra Stone, Kevin M. Lin, Ghanshyam H. Ghelani, Sanik Patel, Sam Benjamin, Stephen Graziano, Leszek Kotula

**Affiliations:** 1 Department of Urology, SUNY Upstate Medical University, 750 East Adams Str., Syracuse, NY 13010, USA; stoneal@upstate.edu (A.S.); linke@upstate.edu (K.M.L.); patesani@upstate.edu (S.P.); 2 Department of Biochemistry and Molecular Biology, SUNY Upstate Medical University, 750 East Adams Str., Syracuse, NY 13210, USA; 3 Department of Hematology/Oncology, SUNY Upstate Medical University, 750 East Adams Str., Syracuse, NY 13210, USA; ghelanig@upstate.edu (G.H.G.); benjamis@upstate.edu (S.B.); grazians@upstate.edu (S.G.); 4 Upstate Cancer Center, SUNY Upstate Medical University, 750 East Adams Str., Syracuse, NY 13010, USA

**Keywords:** AR, TNBC, CDK4/6, CYP17 lyase, PI3K/AKT, ER, PR, HER2, DHT

## Abstract

**Simple Summary:**

Triple negative breast cancer (TNBC) comprises 10–20% of diagnosed breast cancers. TNBCs are devoid of common biomarkers such as an estrogen receptor (ER), progesterone receptor (PR), and human epidermal growth factor receptor 2 (HER2). Research is currently being conducted to determine the androgen receptor’s (AR) role in TNBC and determine its ability to be utilized as an effective drug target in the absence of the commonly targeted receptors. Many studies combine anti-androgen drugs with other chemotherapy to decrease tumor growth and proliferation. The further understanding of AR’s mechanism in tumor cells can improve drug efficacy as well as the prognoses of patients suffering from TNBC.

**Abstract:**

To assess AR’s role in TNBC treatment, various existing and completed clinical trials targeting AR or co-targeting AR with other pertinent signaling molecules were analyzed. Cyclin-dependent kinase 4/6 (CDK4/6), cytochrome P450 17α-hydroxylase/17,20-lyase (CYP17 lyase), and the phosphatidylinositol 3-kinase (PI3K)/protein kinase B (AKT) signaling pathway were some of the most prevalent biomarkers used in combination therapy with AR inhibitors in these trials. Studying how AR functions in tandem with these molecules can have increasing breakthroughs in the treatment options for TNBC. Previous studies have been largely unsuccessful in utilizing AR as the sole drug target for systemic targeted treatment in TNBC. However, there is a lack of other commonly used drug target biomarkers in the treatment of this disease, as well. Thus, analyzing the clinical benefit rate (CBR) within clinical trials that use combination therapy can prove to be imperative to the progression of improving treatment options and prognoses.

## 1. Introduction

Breast cancer is one of the most common cancers in women, yet metastatic breast cancer proves to be the least curable. Specifically, TNBC, devoid of commonly targeted receptors, is an aggressive type that is difficult to treat. Additionally, breast-cancer-related deaths are largely related to metastasis [1]. The current treatment management protocol for TNBC is dependent on the programmed death-ligand 1 (PD-L1) and BReast CAncer gene 1/2 (BRCA1/2) status. If patients are PD-L1-positive, then a combination therapy of pembrolizumab plus chemotherapy is initiated [2]. If patients are PD-L1-negative, but exhibit germline BRCA1/2 mutations, olaparib therapy is initiated [3]. To treat those TNBC cases that are PD-L1- and BRCA1/2-negative, it is urgent to uncover more information regarding the related receptors and proteins that can act as drug targets, one such receptor being AR and its associated signaling pathways. AR’s specific role in breast cancer is currently widely unknown and its association to signaling pathways such as CDK4/6, CYP17 lyase, and PI3K can be a huge development in the treatment of TNBC.

Given CDK4/6’s potent activity in breast cancer cells, researchers aimed to inhibit this activity in conjunction with AR inhibitors [4]. Some studies are combining CDK4/6 inhibitors with AR modulators, the logic behind this being that when ER is positive, AR activation seems to develop tumor suppression activity [5]. If these treatments are showing success in other cancer cell lines, it is imperative to test them on TNBC cell lines as well.

CYP17 lyase inhibitors have been studied extensively in the context of prostate cancer. Many studies have sought its effects specifically in castration-resistant prostate cancer to improve overall survival [6,7,8]. New trials are aiming to apply this mechanistic effect on breast cancer patients, specifically using drugs that inhibit androgens via the CYP17 lyase pathway [9,10].

Similarly, most AR-targeted drugs are approved in the context of prostate cancer, but are still being studied extensively in the context of breast cancer [11]. A combination with PI3K inhibition seemed to catalyze the effects and is another important pathway to research in the ongoing search for effective drugs for TNBC patients [12]. The mechanisms behind these combination drug targets are constantly being further studied to improve treatment. Without this knowledge, the metastasis of breast cancer, in TNBC specifically, will continue to cause death among patients enduring this disease.

## 2. TNBC Subtype of Breast Cancer

TNBC is the deadliest subtype of breast cancer comprising 10–20% of all and characterized by its metastatic phenotype [13]. The majority of TNBC tumors are basal-like, with a reported 60–90% overlap between the subgroups, but the terms TNBC and basal-like are not interchangeable. Basal-like tumors are characterized by genes present in normal breast myoepithelial cells such as cytokeratins CK 5, CK17, P-cadherin, nestin, caveolin 1–2, CD44, and EGFR and have an increased incidence of p53 and BRCA1 mutations, which results in high genomic instability, tumor aggressiveness, and poor prognosis [14]. Basal-like tumors appear at an early age, having a large tumor size and high histological grade. They also have a high mitotic index and the pattern of metastatic relapse is aggressive, which mainly occurs in visceral organs such as the lungs, central nervous system, and lymph nodes.

The androgen receptor is a novel target in TNBC. “Luminar AR” (LR) is a subtype of TNBC with a better prognosis but resistance to neoadjuvant chemotherapy. Seminal studies by Lehman et al., 2011, defined four molecular subtypes of TNBC based on their transcriptional profiles: two basal subtypes (BL1 and BL2), a mesenchymal (M) subtype lacking immune cells, and a luminal androgen receptor subtype (LAR) that is enriched in AR expression and follows its transcription program [15].

## 3. Why Target Androgen Receptor?

The initial results from clinical trials indicated a potential clinical benefit for targeting the AR pathway in breast cancer [12,16]. Retrospective studies in patients demonstrated that LAR tumors are less responsive to standard chemotherapy than other TNBC tumors, underscoring the need for the identification of novel therapeutic strategies [16,17].

AR is expressed in normal breast tissue and decreases to 10–50% expression in TNBC [18,19]. In prostate tumors, many pertinent pathways involved in signaling are regulated by AR activity. AR activity has also been linked to cell cycle promotion, cell metabolism and growth, and other vital cellular processes [20,21]. Thus, researchers are interested in applying this information to breast cancer studies.

**Figure 1:**
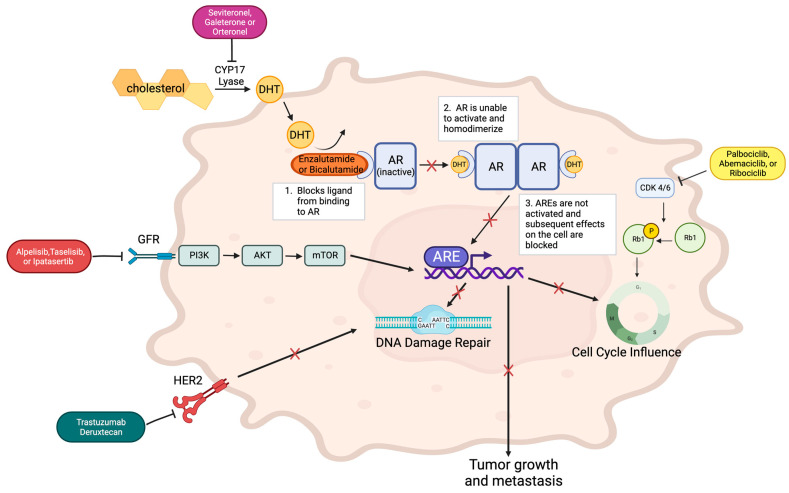
AR is inactive until DHT binds it, allowing for activation via homodimerization. This homodimer binds to androgen response elements (AREs) to contribute to effects on cell cycle, DNA damage repair, and metastasis. The binding action of AR inhibitors, enzalutamide or bicalutamide, blocks the homodimerization of the receptor and its downstream effects. CYP17 lyase inhibitors such as seviteronel, galeterone, or orteronel can augment the effect of AR inhibitors by blocking the CYP17 lyase pathway, which is imperative to forming the DHT that binds and activates AR. PI3K inhibitors can also block expected downstream effects of AR. These inhibitors include alpelisib, taselisib, and ipatasertib. They bind the receptor upstream of the PI3K-AKT-mTOR pathway, which is involved in regulating the growth and proliferation of the cell. CDK4/6 inhibitors such as palbociclib, abemaciclib, and ribociclib block CDK4/6, the kinase responsible for phosphorylating Retinoblastoma (Rb) tumor suppressor gene, which regulates the cell cycle. When this kinase is inhibited, cell cycle regulation is altered, specifically, the checkpoint between the G1 and S phase, amplifying the effects of AR inhibitors on the cell cycle. Trastuzumab deruxtecan inhibits the HER2 receptor, which diminishes its normal impact on DNA damage repair.

AR can also be utilized as a target and possibly be co-targeted with the other signaling molecules it often interacts with, such as CDK4/6, CYP17 lyase, and the PI3K/AKT signaling pathway, as seen in Figure 1. Some AR expression is beneficial for prognosis, too much expression, however, along with heightened activity, leads to enhanced tumor growth [22]. AR’s effects on tumor growth are outlined by the different subtype being studied and the implicated pathways. In ER (−), HER 2 (+) breast cancer, AR transcriptional activity is promoted which increases tumor growth. In TNBC, a similar effect is observed. In breast cancer tumors with ER expression, AR and ER regulate each other’s transcription, and the ratio between the two receptors determines the outcome [22]. AR works in collaboration with many other proteins and pathways, making it an important biomarker for targeting and co-targeting.

### AR in the Context of Hormone Dysregulation

AR is activated by androgens such as dihydrotestosterone (DHT), while its inactive form is bound to heat shock proteins [20]. AR captures androgen in the cytoplasm and is subsequently activated to transport to the nucleus and bind to DNA, promoting the transcription of certain genes affecting cell cycle progression such as MYB or CCND1 [23]. The role of AR in females is still not well understood. In prostate cancer, the interaction between AR activity and the signaling pathways it regulates, such as the PI3K and RAS pathways, is well known. AR dysregulation serves as a signal for hormonal disorders such as androgen insensitivity and prostate cancer. For many cases, androgen deprivation therapies have proven successful, as the activation of its signaling promotes tumor growth [20]. In breast cancer, the role is not as unambiguous. For example, in cases where ER is present, AR has exhibited tumor suppressor activity, and selective AR modulators are proving to be more effective treatment options as compared to AR inhibitors [23].

## 4. Why Co-Target AR with Other Pathways?

AR alone has thus proven unsuccessful in acting as a target for treatment. Synthetic steroidal androgens have caused unwanted adverse effects in treatment. The most effective treatment plan includes agents that target AR combined with signal transduction inhibitors [22]. This is being tested in clinical trials administering AR antagonist bicalutamide in combination with Palbociclib, a CDK4/6 inhibitor [4]. Other treatments that are currently being tested are combining AR antagonists that utilize different mechanisms to target and block AR in AR-positive TNBC. This combination is advantageous in affecting AR and its corresponding genes from different angles, increasing the efficacy of the treatment [24]. AR-positive tumors with ER alpha show better prognosis due to the antagonistic nature of AR and ER alpha, in relation to androgen binding. This competition increases the probability of apoptosis. In contrast, in ER-negative tumors, AR mostly binds to androgens, increasing tumor growth [22]. Combining biomarkers and understanding the mechanism of how they act synchronously is the next step in efficiently treating breast cancer.

### 4.1. Co-Targeting AR and CDK4/6

Clinical trials are currently working on determining the safety and efficacy of combination therapy, including CDK4/6 inhibitors (abemaciclib, ribociclib, and palbociclib), with anti-androgens such as bicalutamide and with selective AR modulators such as enobosarm. The objective of these studies is to understand the relationship between CDK4/6 and AR and why co-targeting these two important biomarkers can improve CBRs among patients. CDK4/6 inhibitors act to prevent the cell cycle from progressing from the G1 to the S phase [25]. AR’s role in nuclear localization may be involved in this progression. AR is considered an oncogene in prostate cancer, thus, inhibiting AR can deter cell proliferation, allowing for tumor suppression [26]. CDK4/6 inhibitors alone have shown non-selective effects [27]. Combining this therapy with AR inhibitors may localize the effects and improve the prognosis for patients undergoing treatment.

Abemaciclib alone has been approved for the treatment of hormone receptor-positive (HR+) advanced/metastatic breast cancer. Compared to the other noted CDK4/6 inhibitors, abemaciclib shows increased potency and specificity for CDK4 based on previous studies [28]. This drug treatment was shown to achieve a CBR of 49%. This was measured by summing complete and partial responses as well as stable disease at or after 24 weeks [29]. This inhibitor is now being tested for TNBC in combination with bicalutamide, as seen in clinical trial NCT05095207, outlined in Table 1. It is hypothesized that the combination of the CDK4/6 inhibitor and AR inhibitor can improve the efficacy of treatment and the overall prognosis for TNBC patients.

Bicalutamide trials combined with ribociclib are currently ongoing. Clinical trial NCT03090165, summarized in Table 1, is investigating the MTD and CBR of the combined treatment. It was hypothesized that the use of CDK4/6 inhibition would enhance the activity of the androgen inhibitor, bicalutamide, as seen in previous prostate cancer cell lines. Current studies using AR inhibitors alone provide modest response rates indicating impending anti-androgen resistance. Thus, combining CDK4/6 inhibition with anti-androgens can eliminate this possible setback. Preliminary results suggest the toleration of the combination drug therapy [30]. If there is continued low toxicity and increased CBR compared to the treatments alone, this combined therapy can be an improvement in the examination of AR+ TNBC treatment availabilities.

Enzalutamide has shown greater affinity for the AR target as compared to bicalutamide. In trials of castration-resistant prostate cancer patients, enzalutamide reduced the risk of progression or mortality by 76% compared to bicalutamide [31]. Studies analyzing AR+ cell lines showed enhanced effects on survival inhibition with enzalutamide treatment combined with ribociclib. When studying growth inhibition via a cell viability assay, enzalutamide treatment combined with ribociclib showed a statistically significant improvement in its ability to diminish the cell viability of AR+ BC cell lines in comparison to enzalutamide alone [32]. The next step is to combine these treatments for a clinical trial to determine the maximum tolerated dose (MTD) of each, as well as progression free survival (PFS) or CBR.

Bicalutamide has shown efficacy alone in treating AR+ BC with a median PFS of 12 weeks [33]. In previous studies, Palbociclib has reduced the growth of AR+ TNBC cells as well, due to this cell type expressing intact retinoblastoma, a common protein target of Palbociclib [34,35]. The clinical trial labeled NCT02605486 in Table 1, tested this AR inhibitor and CDK4/6 inhibitor in combination, hypothesizing that the combined effects would have increased efficacy in AR+ BC patients [33]. The preliminary results show that eleven patients enrolled in the study presented PF for 6 months with no toxicity, indicating the further study of the combined therapy [33].

CDK4/6 inhibition is also being utilized in combination with selective AR agonists such as enobosarm in AR+ ER+ HER2- BC. Given that the presence of ER correlates with an upregulation of AR’s tumor suppressor function, trial NCT05065411 (Table 1) will target CDK4/6 to inhibit cell growth and proliferation while modulating AR’s activity to induce tumor suppression. Enobosarm exerted its effects more efficiently when patients had AR nuclear staining ≥40% and prior CDK4/6 inhibition therapy [5]. This further elaborates on the intricate role of AR in females, and specifically, its relationship with ER in BC cell types beyond TNBC.

### 4.2. Co-Targeting AR and CYP17 Lyase

Androgen receptor signaling has been targeted by directly inhibiting AR, or by decreasing the adrenal and tumoral synthesis of androgens. Androgen production is highly dependent on the enzymatic activity of CYP17, which has both 17α-hydroxylase and 17,20-lyase activity [36]. As the inhibition of hydroxylase activity reduced cortisol levels (and necessitating the co-administration of glucocorticoids with first-generation CYP17 inhibitors), new CYP17 inhibitors (such as seviteronel, galeterone, and orteronel) were developed with the intention of selectively inhibiting CYP17 lyase activity [37]. By blocking androgen synthesis via inhibiting CYP17 lyase activity, tumors have reduced activation of AR signaling. Interestingly, several CYP17 lyase inhibitors such as seviteronel, but not orteronel, have also been shown to have competitive AR inhibition [37]. Seviteronel was able to not only directly bind AR and prevent androgen-mediated gene expression, but also reduce the nuclear translocation and accumulation of AR [37]. Orteronel was not found to have any AR antagonism activity nor ability to attenuate the androgen-mediated nuclear localization of the receptor [37].

Recent phase I and II clinical trials have studied the effects of seviteronel and orteronel in both HR-sensitive breast cancers and TNBCs. Following in vitro studies showing that seviteronel was able to inhibit AR+ TNBC growth on soft agar [38], the CLARITY-01 phase I/II trial (NCT02580448) investigated the effects of seviteronel in both ER+ and TNBCs. The study showed that seviteronel was well tolerated and demonstrated a CBR at 16 weeks of 33% (two out of six patients) in patients with TNBCs and a CBR at 24 weeks of 18% (2 out of 11 patients) in patients with ER+, HER2- metastatic breast cancer [39]. Seven out of ten patients with circulating tumor cells (CTCs) at the baseline had a median reduction in CTCs of −94.3% (range: −27.5% to −100%) [39]. Four patients in the TNBC cohort and eight patients with ER+ metastatic breast cancer remain on seviteronel therapy [39]. These results seem to suggest that seviteronel may be a therapeutic option for patients with AR+ breast cancer.

An early phase 1b clinical trial showed that orteronel was well tolerated in postmenopausal women with HR+ metastatic breast cancer [9]. Of a cohort of eight heavily pretreated patients, a clinical benefit was seen in two patients who had a stable disease for more than 6 months. However, a follow-up phase II trial of orteronel in AR+ metastatic TNBCs or AR+ metastatic HR+ breast cancer showed limited clinical activity [40]. Just 1 patient out of 21 patients (4.8%) with AR+ TNBCs demonstrated an objective response to therapy and none of the 23 patients with AR+ HR+ metastatic breast cancer responded to orteronel [40]. This difference in the patient response to treatment between the seviteronel trial and the orteronel trial seems to suggest that the inhibition of CYP17 lyase alone is not sufficient for therapy. Seviteronel appears to be a much more promising therapy for AR+ TNBCs, potentially due to its ability to both inhibit CYP17 lyase as well as its direct AR inhibition.

Seviteronel has also been shown to work in unison with enzalutamide to radiosensitize AR-positive TNBC tumors more efficiently than when administered individually [41]. This is potentially due to the fact that while seviteronel has direct AR inhibition, its mechanism of AR inhibition is distinct from that of enzalutamide [37]. Given the promise of seviteronel in its phase II trial, and the effects demonstrated in pre-clinical studies in conjunction with enzalutamide, further efforts should investigate the potential effects of dual enzalutamide and seviteronel therapy to co-target AR and CYP17 lyase.

### 4.3. Co-Targeting AR and PI3K/AKT Pathway

Other clinical trials are targeting the PI3K/AKT/mechanistic target of the rapamycin (mTOR) pathway via drugs such as alpelisib, taselisib, and ipatasertib in combination with AR inhibitors, such as enzalutamide. The PI3K/AKT/mTOR pathway—a central pathway regulating cell growth and proliferation—is associated with endocrine resistance in breast cancers [42], and is among the most frequently mutated pathways in TNBCs [43]. This endocrine resistance could be due to reciprocal feedback loops and crosstalk between AR-PI3K pathways [44]. It has been demonstrated in prostate cancer that the inhibition of PI3K leads to the activation of AR, while the loss of AR activity promotes PI3K/AKT pathway activity [21]. Preclinical studies in AR+ TNBC cell lines have also shown that treatment with AR inhibitors along with PI3K/mTOR inhibitors was more effective than treatment with AR inhibitors alone [43].

Following the promising preclinical results, a recent phase Ib/II clinical trial investigated the safety and efficacy of enzalutamide in combination with taselisib, a PI3K inhibitor [12]. In phase I, it was discovered that the combination was well tolerated among patients [12]. Nineteen TNBC patients were enrolled for phase II, with 5 patients receiving only enzalutamide and 14 receiving enzalutamide with 4 mg of taselisib. At 16 weeks, it was found that one patient receiving the combination therapy had a partial response, while four patients had stable diseases. The CBR at 16 weeks for the combination therapy was 35.7%, compared to 0% for the enzalutamide-only arm [12]. The median PFS was 3.4 months for patients with both taselisib and enzalutamide [12]. These results appear to suggest that co-targeting AR and PI3K pathways can cause a clinical benefit compared to targeting AR alone.

The DESTINY-Breast04 trial used trastuzumab deruxtecan in patients with HER-2-low TNBC tumors. HER-2-low was defined as a 1+ score on immunohistochemical analysis (IHC) or 2+ IHC with a negative in situ hybridization result [45]. The ASCENT trial used sacituzumab govitecan in patients with tumors expressing human trophoblast cell-surface antigen 2 (Trop-2), which is expressed in many breast cancers [46]. The BEGONIA trial used datopotamab deruxtecan plus durvalumab in patients with Trop-2 (+) and PD-L1 (+) tumors [47]. All outcomes seen in the table were compared to the physician’s choice of chemotherapy.

### 4.4. HER2 Low and Trastuzumab Deruxtecan

Some TNBC cells may not be completely HER2 negative, rather, they have extremely low HER2 level expression [48]. In fact, over half of those cancers that are considered HER2-negative actually express low levels of the receptor. Current chemotherapy agents directed toward targeting HER2 have not proven to be effective in treating this subcategory [45]. Trastuzumab deruxtecan, being among the few effective agents, can target HER2, even at low expression levels, via the anti-HER2 monoclonal antibody portion of the agent and subsequently destroy the affected cells via the topoisomerase I inhibitor portion of the agent [45]. The drug presented promising preliminary antitumor activity results in the trial [49] (Table 2). This type of innovative drug treatment should be further researched for application to anti-AR therapies as well.

## 5. Conclusions

AR and current co-targets have been thoroughly explored throughout this paper, highlighting potential treatment options for patients with AR+ TNBC. Thus far, major co-targets include CDK4/6, CYP17 lyase, and the PI3K/AKT pathway. There are many ongoing clinical trials targeting both AR along with these biomarkers and determining the efficacy of treatment, measured in CBR and PFS, as well as the tolerance of combination therapy, measured in MTD.

Future studies can continue the trend of utilizing prostate cancer treatment targets in the study of breast cancer, specifically TNBC. These biomarkers can be co-targeted with AR to improve the effectiveness of the treatment. One possible co-target is the MEK/ERK pathway. Prostate cancer studies have already demonstrated that the IgG1 heavy chain is elevated and when knocked down, there is a downstream inhibition of the MEK/ERK/c-myc pathway [50]. This indicates its role as a possible target for chemotherapy treatment. In breast cancer, sulforaphane, a natural compound, was found to inhibit MEK and ERK phosphorylation, demonstrating a potential to halt cell invasion and migration [51]. Further research needs to be conducted to confirm that this treatment can be beneficial or targeted in combination with a biomarker such as AR. Another potential co-target is ABI1, which acts as an oncogene in breast cancer cells and is associated with aggressive phenotypes [52]. ABI1 was recently shown to regulate the transcriptional activity of AR in prostate cancer cells and can prove to be a novel and effective drug target for AR+ TNBC cells [53]. In addition to increasing potential future co-targets, treatment can have more widespread effects if administered to TNBC patients with low AR expression as opposed to high AR-expressing tumors only. Trastuzumab targets HER2-positive breast cancer cells, which is commonly defined as those cells with a high expression of HER2. Recent studies demonstrated that Trastuzumab was found to have noteworthy antitumor activity in breast cancer cells with a low expression of the HER2 receptor as well [49]. Thus, we can use this paradigm to treat TNBC cells with a low AR expression in a similar manner.

The current clinical trials highlighted here, along with these new potential co-targets and treatment options have exceptional potential and may prove to change the distressing prognosis TNBC patients currently live with.

## Figures and Tables

**Table 1:** Current clinical trials co-targeting AR and CDK4/6, CYP17 lyase, and PI3K/AKT pathway.

Trial Number	Trial Name	Drug Name(s)	Purpose	Outcomes	Phase
NCT05095207	Abemaciclib in combination with bicalutamide for AR+, HER2-metastatic breast cancer	Abemaciclib and Bicalutamide	Determine the dose-limiting toxicity and efficacy of this combination therapy	Accrual Status: Active Hypothesized that the two drugs together will improve CBR based on preclinical data and the drug properties	IB/II
NCT03090165	Ribociclib and bicalutamide in AR+ TNBC	Ribociclib and Bicalutamide	Determine the safety and efficacy of this combination therapy	Accrual Status: Active Primary outcome to be measured by MTD for combination of drugs without dose-limiting toxicity and CBR	I/II
NCT02605486	Palbociclib in combination with bicalutamide for the treatment of AR+ metastatic breast cancer (MBC)	Palbociclib and Bicalutamide	Determine the safety and efficacy of this combination therapy as well as determining effective dosage	Accrual Status: Active Primary outcomes to be measured by determination of the RP2D (phase I) and PFS (phase II)	I/II
NCT05065411	Efficacy and safety evaluation of enobosarm in combo with abemaciclib in treatment of ER+ HER2-metastatic breast cancer	Enobosarm and Abemaciclib Combo	Determine the safety of enobosarm used with abemaciclib as compared to the estrogen-blocking control group, exemestane, and fulvestrant	Accrual Status: Active Primary outcome to be measured by PFS in the combination drug group as compared to the control group	III
NCT01808040	A Phase IB Study of TAK-700 in postmenopausal women with HER2+ metastatic breast cancer	TAK700 (CYP17 Lyase Inhibitor)	Determine dose escalation and dose expansion of TAK700 in female metastatic breast cancer patients, test TAK700′s ability to decrease estrogen levels	Accrual Status: Completed Results not yet submitted, primary outcomes to be measured by number of patients with adverse effects to determine RP2D, decrease in serum estradiol levels	I
NCT02130700	Oral VT-464 in patients with castration-resistant prostate cancer (CRPC) previously treated with enzalutamide, AR+ TNBC patients and men with ER+ breast cancer	VT-464 (lyase-selective inhibitor of CYP17)	Determine the efficacy and safety of VT-464 in CRPC patients treated with enzalutamide, AR+ TNBC patients, and male AR+ BC patients	Accrual Status: Completed Results not yet posted, primary outcomes to be measured by serum PSA decrease, PFS, and CBR	II
NCT02580448	CYP17 Lyase and AR inhibitor treatment with seviteronel trial	Seviteronel	Determine the RP2D of seviteronel for TNBC or ER+ BC patients in phase I as well as safety, pharmacokinetics, and pharmacodynamics in phase II	Accrual Status: Completed Results not yet submitted, primary outcome to be measured by CBR at 16 weeks for TNBC female patients and all male patients, CBR at 24 weeks for ER+ BC female patients	I/II
NCT02457910	Taselisib and enzalutamide in treating patients with AR+ triple-negative metastatic breast cancer	Taselisib and Enzalutamide	Determine the side effects and most effective dose of this combination therapy in AR+ TNBC	Accrual Status: Active 0.357 CBR in combination therapy patients, dose limiting toxicity of 0 measured in doses ranging 2–8 mg of taselisib with 160 mg enzalutamide	IB/II
NCT03207529	Alpelisib and enzalutamide in treating patients with AR+, PTEN+ metastatic breast cancer	Alpelisib and Enzalutamide	Determine the maximum tolerated dose (MTD) of this combination therapy	Accrual Status: Active Primary outcome will be measured using MTD	I
NCT03840200	A study evaluating safety, pharmacokinetics, and efficacy of ipatasertib administered in combination with rucaparib in participants with advanced breast, ovarian, and prostate cancer	Ipatasertib and Rucaparib	Determine safety and pharmacokinetics of this combination therapy as well as dose escalation and expansion	Accrual Status: Completed Results not yet submitted, primary outcome to be measured by percentage of patients with adverse effects and dose-limiting toxicities	I

**Table 2:** Examples of antibody drug conjugates (ADC) under clinical trials for TNBC.

Trial Name	Objective Response Rate (%)	Progression-Free Survival (Months)	Overall Survival (Months)	Tumor Background
DESTINY-Breast04 (trastuzumab deruxtecan)	52.3	9.9 HR * = 0.50; *p* < 0.001	23.4 HR = 0.64; *p* = 0.001	HER-2 low
ASCENT (sacituzumab govitecan)	35	5.6 HR = 0.41; *p* < 0.001	12.1 HR = 0.48; *p* < 0.001	Trop-2 (+)
BEGONIA (Ib/II) (datopotamab deruxtecan plus durvalumab)	79	13.8	Not measured	Trop-2 (+) PD-L1 (+)

* Hazard Ratio.
